# A haplotype-resolved triploid genome assembly of *Zingiber striolatum* sheds light on genome expansion and trait evolution in Zingiberaceae

**DOI:** 10.1093/hr/uhag214

**Published:** 2026-06-03

**Authors:** Zhi-Feng Chen, Wei-Hong Sun, Li-Yao Su, Guo-Fei Tan, Yu Lu, Lian Luo, Wen-Hui Li, Chun-Xiao Li, Zi-Wei Tan, Zhuang Zhao, Chang-Ling Sui, Zhong-Jian Liu, Ai-Sheng Xiong

**Affiliations:** College of Ecology, Zunyi Normal University, Zunyi 563006, China; State Key Laboratory of Crop Genetics & Germplasm Enhancement and Utilization, Ministry of Agriculture and Rural Affairs Key Laboratory of Biology and Germplasm Enhancement of Horticultural Crops in East China, College of Horticulture, Nanjing Agricultural University, Nanjing 210095, China; College of Ecology, Zunyi Normal University, Zunyi 563006, China; State Key Laboratory of Crop Genetics & Germplasm Enhancement and Utilization, Ministry of Agriculture and Rural Affairs Key Laboratory of Biology and Germplasm Enhancement of Horticultural Crops in East China, College of Horticulture, Nanjing Agricultural University, Nanjing 210095, China; Institute of Horticulture, Guizhou Academy of Agricultural Sciences, Guiyang 550025, China; College of Ecology, Zunyi Normal University, Zunyi 563006, China; College of Ecology, Zunyi Normal University, Zunyi 563006, China; China Strait Horticulture Technology Innovation Hub, College of Landscape Architecture and Art, Fujian Agriculture and Forestry University, Fuzhou 350002, China; College of Ecology, Zunyi Normal University, Zunyi 563006, China; College of Ecology, Zunyi Normal University, Zunyi 563006, China; College of Ecology, Zunyi Normal University, Zunyi 563006, China; College of Ecology, Zunyi Normal University, Zunyi 563006, China; China Strait Horticulture Technology Innovation Hub, College of Landscape Architecture and Art, Fujian Agriculture and Forestry University, Fuzhou 350002, China; State Key Laboratory of Crop Genetics & Germplasm Enhancement and Utilization, Ministry of Agriculture and Rural Affairs Key Laboratory of Biology and Germplasm Enhancement of Horticultural Crops in East China, College of Horticulture, Nanjing Agricultural University, Nanjing 210095, China

## Abstract

The genus *Zingiber* represents an economically important group within Zingiberaceae, distinguished by its aromatic and bioactive rhizomes. *Zingiber striolatum* Diels is an endemic Chinese medicinal and edible plant highly valued for its flower buds, which are rich in natural red pigments (yanghe red pigment, YRP). Here, we present the first haplotype-resolved, chromosome-scale assembly of ‘Ziyanghe,’ a purplish-red-bud cultivar widely grown in Guizhou Province. The triploid genome was resolved into three haplotypes: subA (2.80 Gb), subB1 (2.66 Gb), and subB2 (2.64 Gb), with the latter two exhibiting strong collinearity. Comparative analysis across five Zingiberaceae genomes revealed a family-specific whole-genome duplication ~ 52.5 million years ago (Mya) and lineage-specific bursts of long terminal repeat retrotransposons occurring between 0.15 and 0.66 Mya. These events likely contributed to substantial genome expansion in *Z. striolatum*. Evolutionary analysis of gene families suggested parallel subgenome adaptation to montane shade environments and rhizome-specific volatile biosynthesis. Metabolomic profiling identified cyanidin-3-O-glucoside, peonidin-3-O-glucoside, delphinidin-3-O-glucoside, and delphinidin-3-O-rutinoside as the major YRP components, with quercetin-3-O-glucoside functioning as a co-pigment. Integrated multi-omics analysis provides a preliminary molecular framework for YRP biosynthesis. This high-quality genomic resource significantly enhances phylogenomic understanding of Zingiberaceae and provides a foundation for the genetic improvement and metabolic engineering of anthocyanin biosynthesis in this species.

## Introduction

Zingiberaceae, with its center of diversity and origin in tropical Asia, comprises ~1600 species distributed across 50 genera [1]. Within this family, the genus *Zingiber* encompasses more than 150 species globally, 42 of which are distributed in China. Distinguished by prostrate or tuberous rhizomes rich in aromatic and pungent secondary metabolites, these plants are widely used in cuisine and traditional medicine [2, 3]. *Zingiber striolatum* Diels, commonly known as ‘yanghe’, is a perennial rhizomatous herb native to the mountainous regions of southern China and Southeast Asia [4, 5]. It thrives in shaded, humid habitats such as forest understories and stream margins at 300–1900 m elevation [6]. Owing to its nutritional composition and biological activities, *Z. striolatum* has become a focal point of research in food science, agriculture, and nutrition; its cultivation is now primarily concentrated in Southwest China, specifically in Guizhou and Yunnan Provinces [7]. As both a vegetable and a medicinal plant, it is consumed primarily for its tender flower buds [8]. The buds contain abundant proteins, dietary fiber, polysaccharides, and several essential amino acids, with levels often higher than those in common cereals [9]. Bioactive constituents, including volatile oils, flavonoids, and natural pigments, contribute to the reported hypoglycemic, hypolipidemic, anticoagulant, antiviral, anti-infective, and anti-aging effects [10, 11]. Essential oils in rhizomes and buds are dominated by the monoterpenes β-phellandrene and β-pinene [12, 13]. Notably, its vividly colored buds contain a unique pigment complex termed yanghe red pigment (YRP) [14], a defining feature with relevance for natural colorant development and health-related bioactivity and whose molecular genetic basis remains to be clarified.

Existing studies suggest that YRP consists mainly of anthocyanins, a subgroup of flavonoids responsible for pigmentation in many plant tissues [15]. Anthocyanins possess strong antioxidant capacity, support human health, and help plants tolerate abiotic stressors, including intense light, UV radiation, and extreme temperatures [16]. Their chromatic expression spans pink to deep burgundy, with pigment type and concentration determining final coloration [17–19]. Among the six anthocyanidin forms commonly detected in plants, cyanidin and delphinidin are prevalent and typically produce red-to-purple or blue-purple shades. Peonidin yields reddish-purple coloration, pelargonidin produces orange-to-red hues that may shift to purple when combined with other pigments, and petunidin and malvidin generate blue-purple tones. Anthocyanin biosynthesis proceeds through the phenylpropanoid–flavonoid pathway [20, 21]. The MYB-bHLH-WD40 repeat (MBW) transcriptional complex further regulates flavonoid pigmentation [22, 23]. In addition to anthocyanins, flavones and flavonols can contribute to coloration through co-pigmentation [24]. To date, anthocyanin biosynthesis in *Z. striolatum* buds has been examined only from partial transcriptome data [15], and neither the precise anthocyanins present nor the contribution of co-pigment flavonoids to coloration has been fully examined.

A complete and accurate reference genome is critical for studies of species origin, evolutionary history, phenotypic trait development, and secondary-metabolite biosynthesis [25]. In Zingiberaceae, chromosome-scale genome assemblies are available for *Zingiber officinale* [26, 27], *Roscoea debilis* [28], *Amomum tsaoko* [29, 30], *Wurfbainia villosa* [31], and *Curcuma longa* [32, 33], providing a basis for reconstructing family-level evolution and for investigating biosynthetic pathways for key metabolites such as gingerols and terpenoids. Here, we present the first haplotype-resolved, chromosome-scale genome of triploid *Z. striolatum* (2*n* = 3 *×* = 33), generated using PacBio high-fidelity (HiFi) long reads, Illumina short reads, and high-throughput chromosome-conformation-capture (Hi-C) sequencing. Comparative analysis across available Zingiberaceae genomes reveals transposable element (TE) bursts and clarifies evolutionary trajectories within the family. Integration of genomic, transcriptomic, and metabolomic data further elucidates the molecular genetics underpinning pigmentation in *Z. striolatum* buds.

## Results

### Chromosome-level assembly and annotation of the *Z. striolatum* genome

The widely cultivated purplish-red-bud *Z. striolatum* (2*n* = 3 *×* = 33) cultivar ‘Ziyanghe’ from Guizhou Province was selected for whole-genome sequencing (Fig. 1a; Fig. S1a). Flow cytometry estimated a genome size of 7.96 Gb (Fig. S1b). To generate a high-quality reference genome, 78.45 Gb of clean PacBio HiFi long reads (~9.6× coverage; read N50 = 17 450 bp) and 325.72 Gb of clean Hi-C data were obtained (Table S1). The preliminary assembly, generated using hifiasm in Hi-C integration mode (−hic), yielded 873 contigs with a total length of 8.15 Gb. The assembly exhibited high continuity, with a contig N50 of 35.21 Mb and a GC content of 41% (Table S2). Hi-C scaffolding anchored 8.09 Gb (99.28%) of the assembly to 33 pseudochromosomes (Table S3). The Hi-C heatmap exhibited strong interaction signals along the diagonal, indicating high continuity and accuracy of the genome assembly (Fig. S2). Smudgeplot profiling revealed that 69% of *K*-mer pairs were AAB-type, compared with 25% AB and 0.06% AAABBB, supporting triploidy in *Z. striolatum* (Fig. 1b). Subgenome partitioning assigned the 33 pseudochromosomes to one A subgenome (subA) and two B subgenomes (subB1 and subB2) (Fig. 1c; Fig. S3). Ths subA assembly was 2.80 Gb (scaffold N50 = 268.18 Mb), subB1 was 2.66 Gb (scaffold N50 = 268.32 Mb), and subB2 was 2.64 Gb (scaffold N50 = 260.32 Mb), with chromosome lengths ranging from 172.64–333.47 Mb, 161.29–305.35 Mb, and 169.79–299.00 Mb, respectively (Table 1; Fig. S3). The haplotype-resolved genome of *Z. striolatum* exceeds the sizes of *Z. officinale* haplotypes A and B (1.56 Gb and 1.40 Gb) [27] and is larger than the triploid genome of *C. longa* (1.11 Gb) [33], but is broadly comparable to diploid *A. tsaoko* (2.08–2.00 Gb) [29, 30] and *W. villosa* (2.8 Gb) [31].

**Figure 1:**
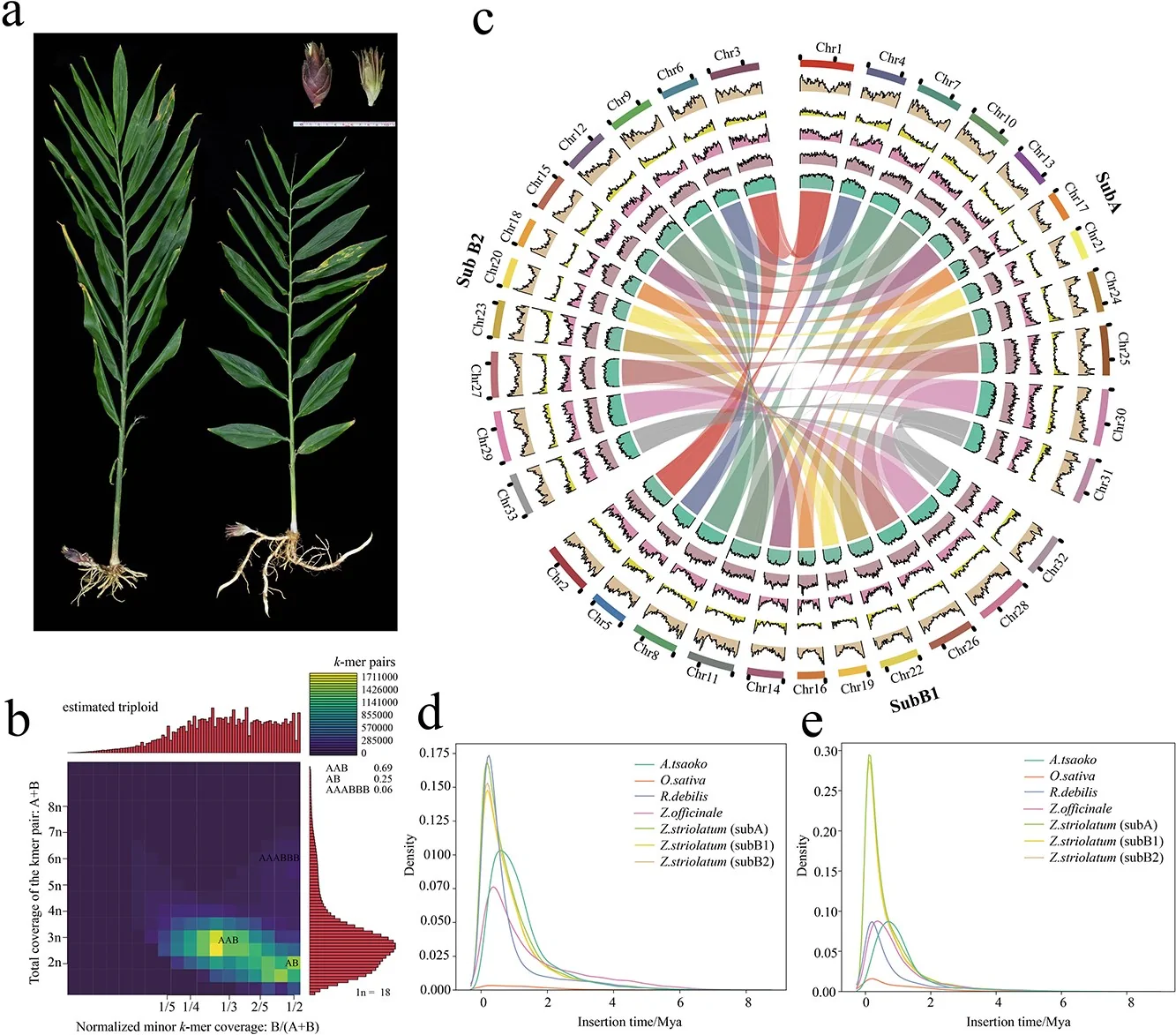
Overview of the *Z. striolatum* genome assembly and features. (a) *Z. striolatum* plants. Left: ‘Ziyanghe’, the widely cultivated variety with purplish-red buds. Right: ‘Baiyanghe’, a natural mutant characterized by white buds. (b) Smudgeplot analysis of the triploid *Z. striolatum* genome. The *y*-axis represents the total coverage of homologous *K*-mer pairs (CovA+CovB), while the *x*-axis indicates the relative minor *K*-mer coverage (CovB/CovA+CovB). (c) Circular visualization of the 33 *Z. striolatum* pseudochromosomes. Tracks from outside to inside are as follows: 33 pseudochromosomes, GC content, gene density, TE density, SNP density, *Copia* density, and syntenic links between homologous chromosome groups. (d and e) Distribution of insertion times for *Copia* and *Gypsy* LTR-RTs in *Z. striolatum* compared with those in four other species: *Z. officinale*, *A. tsaoko*, *R. debilis*, and *O. sativa*.

**Table 1:** Statistics of the *Z. striolatum* genome.

**Item**	***Z. striolatum***	**SubA**	**SubB1**	**SubB2**
Scaffold N50 length (Mb)	261.73	268.18	268.32	260.32
Maximum scaffold length (Mb)	333.49	333.49	305.35	299
Total assembly size (Mb)	8153.22	2801.33	2655.11	2637.73
Assembly BUSCO assessment (%)	98.51	98.02	96.96	97.89
LTR assembly index (LAI)		26.61	24.66	24.83
Number of protein-coding genes	93 286	31 168	30 903	31 160
Annotation BUSCO assessment (%)	97.07	91.2	91.88	91.7
Repeat of genome (%)	89.96	90.32	89.95	89.87
LTR of genome (%)	82.83	83.29	82.85	82.72
LTR-*Copia* of genome (%)	46.8	47.37	46.54	46.52
LTR-*Gypsy* of genome (%)	34.94	34.85	35.24	35.12

The HiFi of this haplotype-resolved assembly was further validated by an exceptionally low post-phasing single nucleotide polymorphism (SNP) rate of 0.0053% overall. This metric remained consistently minimal across all subgenomes (subA: 0.0050%; subB1: 0.0049%; subB2: 0.0062%) (Table S4), demonstrating that the vast majority of heterozygous sites were accurately resolved. Additionally, remapping of PacBio HiFi reads revealed a remarkably uniform depth distribution consistent with the expected sequencing coverage, with no extensive or continuous regions of aberrant depth (Fig. S4). These findings collectively confirm that the three subgenomes were successfully resolved as distinct, high-quality sequences with minimal assembly collapse. Genome completeness reached 98.51% based on 1614 Benchmarking Universal Single-Copy Orthologs (BUSCOs) (Table 1). The completeness scores for the three subgenomes (subA: 98.02%; subB1: 96.96%; subB2: 97.89%) exceeded those of the published *Z. officinale* genome [27]. The calculated long terminal repeat (LTR) assembly index (LAI) values for the three subgenomes were 26.61, 24.06, and 24.83, respectively (Table 1). All values exceeded the ‘Gold Standard’ threshold (LAI > 20) [34]. These results demonstrate high-quality, chromosome-level completeness in the assembled genome.

Gene annotation was performed using *ab initio* prediction, homology-based methods, and RNA-seq support, yielding 93 286 protein-coding genes (Table 1; Fig. S5), of which 92 911 (99.60%) were functionally annotated (Table S5; Fig. S6). SubA, subB1, and subB2 contained 31 168, 30 903, and 31 160 genes, respectively. Gene structure was consistent among subgenomes, with mean gene lengths of 6084.35, 6053.25, and 6018.29 bp, an average of 5.67 exons per gene, and mean coding sequence lengths of 1279–1284 bp (Table S6). The published diploid *Z. officinale* genome contains 39 555 genes with shorter average gene and coding sequence (CDS) lengths (4838 and 1171 bp; mean exons = 5.59) [27]. Thus, the three *Z. striolatum* subgenomes harbor a larger gene repertoire and longer gene structures than the published diploid *Z. officinale* genome (Fig. S5a, b, c, d and e). Gene set evaluation confirmed transcriptomic, homology, and *de novo* support for all annotated genes, with ≥80% overlap (Table S7). BUSCO evaluation of the gene sets yielded completeness values of 91.20%, 91.88%, and 91.70% for subA, subB1, and subB2, confirming high annotation integrity (Table 1).

Repetitive elements were identified through homology-based and *de novo* approaches. A total of 7.33 Gb (89.96%) of the genome comprised repetitive sequences: 2.53 Gb in subA (90.32%), 2.39 Gb in subB1 (89.95%), and 2.37 Gb in subB2 (89.87%) (Table 1; Table S8). Long terminal repeat retrotransposons (LTR-RTs) were dominant, accounting for 82.83% (*Copia*: 46.80%; *Gypsy*: 34.94%), 83.29% (*Copia*: 47.37%; *Gypsy*: 34.85%), and 82.85% (*Copia*: 46.54%; *Gypsy*: 35.24%) of the genome in subA, subB1, and subB2, respectively (Table 1; Table S9). LTR abundance in *Z. striolatum* was markedly higher than in *Z. officinale* (61.37%), *C. longa* (69.4%), *A. tsaoko* (66.49%), and *W. villosa* (66.49%) (Table S10). Comparative analysis of intact LTR insertion times across *Z. striolatum*, *Z. officinale*, *A. tsaoko*, *R. debilis*, and *Oryza sativa* revealed that *Z. striolatum* harbors the highest abundance of intact LTRs (Table S11). Insertion dynamics indicate that *Copia* and *Gypsy* elements in the four Zingiberaceae species experienced synchronized expansion bursts between 0.15 and 0.66 million years ago (Mya) (Fig. 1d and e), suggesting a shared transposition event. The notably higher density of *Copia* and *Gypsy* elements within the three subgenomes of *Z. striolatum* further explains why this species exhibits a greater LTR proportion than its close relatives.

### Genomic variation and chromosome evolution

The three subgenomes of *Z. striolatum* share similar genomic characteristics, including comparable genome sizes, gene numbers, and repeat content (Table 1). Genomic synteny revealed strong similarity across the three homologous chromosome sets, with high gene collinearity (Fig. 1c; Fig. S7). Whole-genome alignment across 11 homologous chromosome groups (Fig. 2a and b; Table S12) identified 7.87 million SNPs and 0.7 million insertions, with fewer variants on chromosome groups 06 and 07 than in the other groups. A total of 684.82 Mb of syntenic regions, 1.03 Gb of highly divergent regions, and 3.3 Gb of non-aligned sequence were detected. Structural variants (SVs) included inversions, translocations, duplications, and deletions, which provide insight into genome evolution and provide potential targets for genetic improvement [35, 36]. Cumulative SV lengths reached 167.79, 169.08, and 176.26 Mb in subA, subB1, and subB2, respectively. Inversions represented 93.51%–94.12% of total SVs, with 43.77%–46.20% concentrated in chromosome group 01 (Table S12). Duplication patterns closely mirrored inversion distributions. Comparative analysis with *Z. officinale* revealed several large inversion blocks, including a 60–130 Mb region on *Z. officinale* Chr01 relative to a 128–280 Mb region on *Z. striolatum* subA1 Chr01 (Fig. 2b). SubA diverged more strongly from subB1, showing dense clusters of inversions, translocations, and duplications across Chr01, whereas homologous chromosomes in subB1 and subB2 were highly conserved, with Chr10 showing the greatest stability.

**Figure 2:**
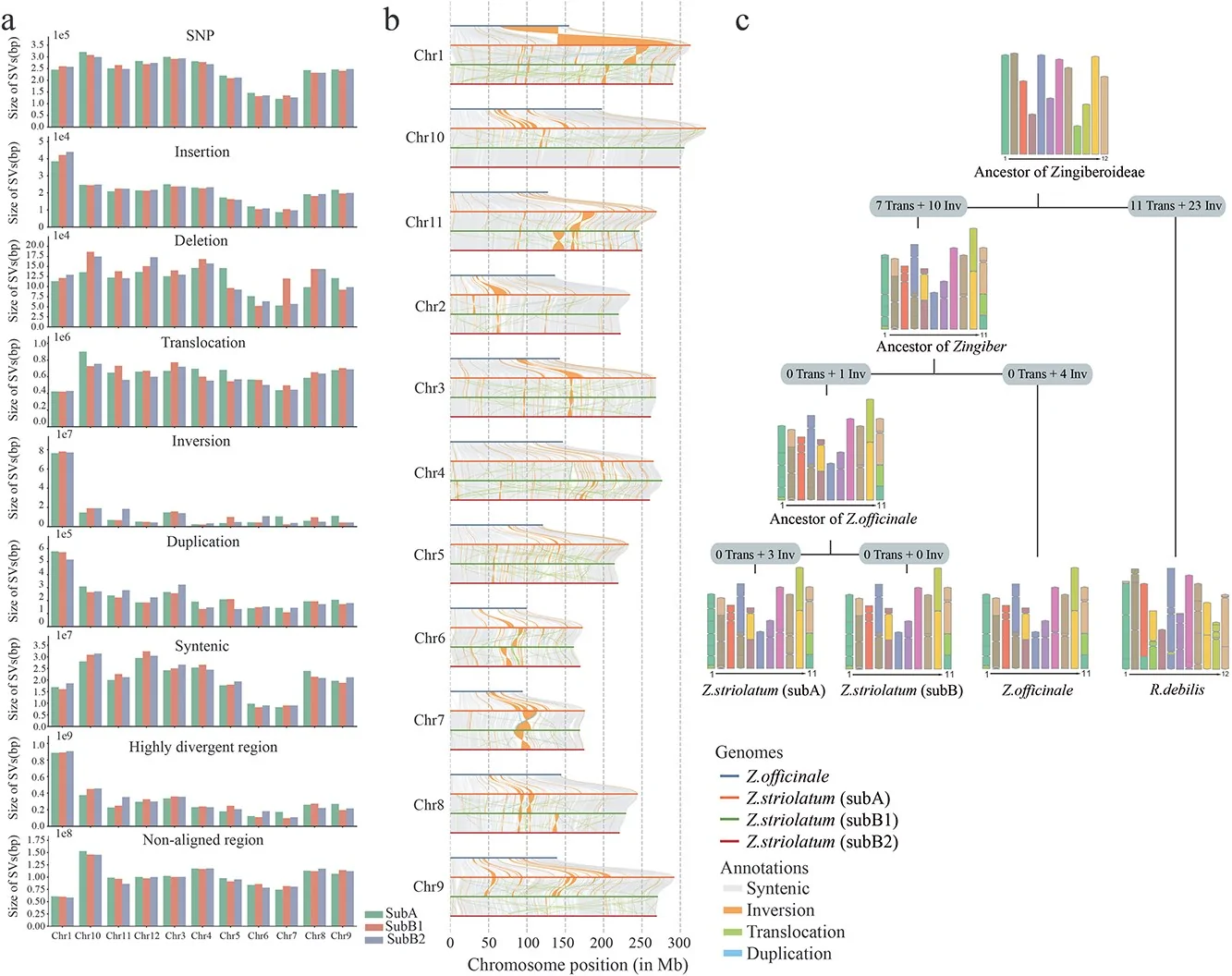
Comparative analysis of eleven homologous chromosome sets among three *Z. striolatum* subgenomes. (a) Distribution of structural variation size among the three *Z. striolatum* subgenomes. (b) Structural rearrangements in homologous chromosome sets between diploid *Z. officinale* and the three *Z. striolatum* subgenomes. (c) Karyotype evolution in *Zingiber*.

To investigate chromosome evolution, we reconstructed ancestral karyotypes of *Zingiber* (Fig. 2c). The putative ancestral Zingiberaceae genome (*x* = 12) diverged into the extant *R. debilis* karyotype through 11 translocations and 23 inversions. In parallel, the same ancestral genome underwent seven translocations, ten inversions, and a fusion event affecting ancestral chromosome 12, forming the proto-*Zingiber* karyotype (*x* = 11). Subsequent one-step and four-step inversion events produced the ancestral karyotypes of *Z. striolatum* and *Z. officinale*, respectively. The ancestral *Z. striolatum* configuration was retained in subB without further rearrangement, whereas subA underwent three paracentric inversions. These results indicate that the chromosomal structure of *Z. striolatum* has remained largely stable since divergence, with its modern genome shaped by relatively limited rearrangements from the ancestral *Zingiber* lineage.

### Triploid formation

Smudgeplot results indicate that *Z. striolatum* is an allotriploid with an AAB-type genomic configuration (Fig. 1b). This was further corroborated by chromosome similarity analysis, which partitioned the assembly into two subgenomes, A and B, with subB comprising two haplotypes (Fig. S3). A 1:3 collinearity relationship between the genomes of diploid relatives (*Z. officinale*, *R. debilis*, *A. tsaoko*, and *W*. *villosa*) and *Z. striolatum* provided additional evidence for its triploidy status (Fig. S8a, 8b, 8c and 8d). To investigate the origin of triploidy, a phylogenetic tree was constructed using 15 angiosperms, including five Zingiberaceae species (Fig. 3a). Our results indicate that the Zingiberaceae and *Musa acuminata* lineages diverged ~76.5 Mya (95% HPD CI: 61.1–90.6 Mya), followed by the diversification of extant Zingiberaceae ~21.3 Mya (95% HPD CI: 14.2–30.7 Mya). Notably, *Z. striolatum* and *Z. officinale* formed a sister clade to *R. debilis*, diverging ~7.3 Mya (95% HPD CI: 4.5–11.1 Mya). Within *Z. striolatum*, the subA and subB lineages were estimated to have diverged ~2.0 Mya (95% HPD CI: 1.3–3.2 Mya) (Fig. 3a), collectively pointing toward an allopolyploid origin. To estimate the timing of the allopolyploidization event, we compared the TE divergence patterns of subA and subB. Their overlapping TE profiles suggest comparable evolutionary rates, while a non-overlapping divergence window (31%–19%) corresponds to the interval between ancestral divergence (~3.2 Mya) and subsequent genome merger (~1.2 Mya) (Fig. 3b).

**Figure 3:**
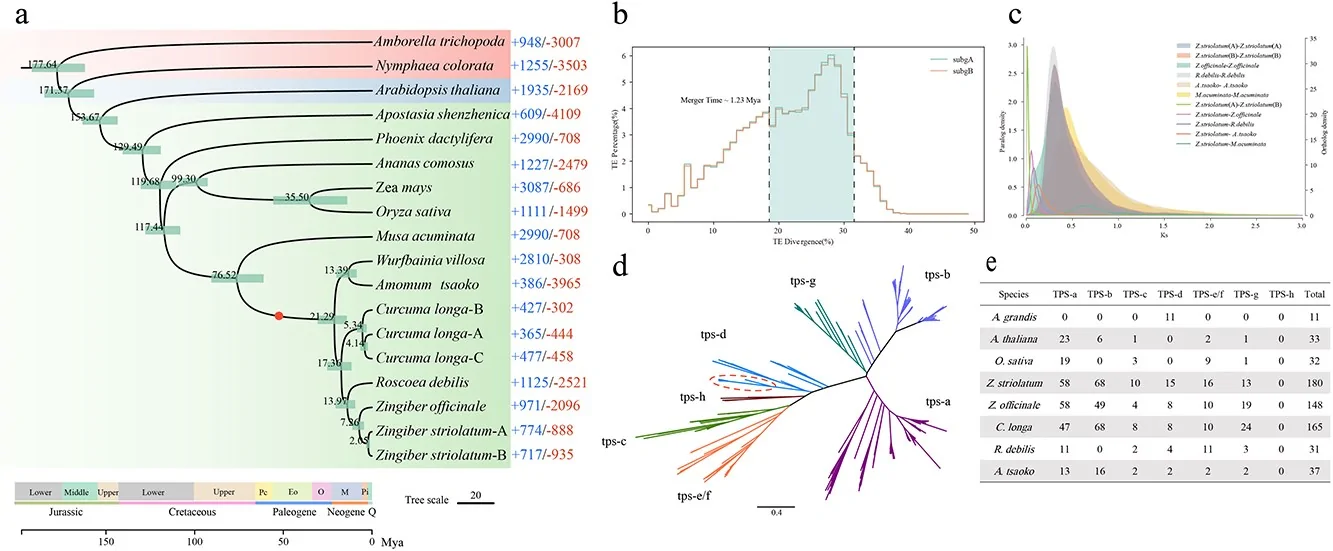
Phylogenetic tree showing divergence times and the evolution of gene family size in *Z. striolatum* and 14 other plants. (a) Estimation of divergence times and gene family expansion and contraction in 15 species. Numerical values at each node represent estimated divergence times (Mya). Circles on the phylogenetic tree indicate the Zingiberaceae-specific WGD event. (b) Distribution of sequence divergence rates of TEs as percentages of subgenome size of *Z. striolatum*. Divergence in TE content between subgenomes A and B indicates diploid ancestor divergence (~3.2 Mya) and subgenome merger (~1.2 Mya) in triploid *Z. striolatum*. (c) Distributions of *Ks* for *Z. striolatum*, *Z. officinale*, *R. debilis*, and *A. tsaoko*. Intraspecific *Ks* peaks signify WGD events, while interspecific *Ks* peaks correspond to speciation events. (d) Phylogenetic relationships of the TPS gene family. Dashed lines highlight members of the Zingiberaceae species TPS-d subfamily. (e) Distribution of TPS subfamily members identified across the surveyed species.

### Whole-genome duplication

Whole-genome duplication (WGD) events are widespread in angiosperms and have played a major role in genome evolution [37]. To assess the WGD history of *Z. striolatum*, synonymous substitution rates (*Ks*) were calculated for paralogous gene pairs from four Zingiberaceae species, including *Z. striolatum*, *Z. officinale*, *R. debilis*, and *A. tsaoko* (Fig. 3c). Paralogous *Ks* distributions across the four species exhibited a shared peak at ~0.3, consistent with an ancestral WGD event. The orthologous *Ks* peaks between *Z. striolatum* and its relatives (*Z. officinale*, *R. debilis*, and *A. tsaoko*) were identified at ~0.05, 0.08, and 0.13, respectively. Because these divergence peaks all occurred after the *Ks* = 0.3 event, the WGD predates the diversification of these lineages. Furthermore, comparisons with *M. acuminata* showed an orthologous *Ks* peak value > 0.03, suggesting that the shared WGD occurred after the divergence of Zingiberaceae from *M. acuminata* (~75.6 Mya; 95% HPD CI: 61.1–90.61 Mya) (Fig. 3a). Accordingly, we infer that this Zingiberaceae-specific WGD event occurred ~52.5 Mya.

### Gene family evolution

Lineage diversification and phenotypic innovation in plants often reflect gene family expansion and contraction [38]. Comparative genomic analysis of *Z. striolatum* and 14 additional species identified 831 expanded and 534 contracted gene families in the ancestor of *Z. striolatum*, 774 expanded and 888 contracted gene families in subA, and 717 expanded and 935 contracted gene families in subB (Fig. 3a; Table S13; Fig. S9). Both subgenomes exhibited significant expansion in pathways associated with phenylpropanoid (ko00940) and monoterpenoid (ko00902) biosynthesis (Fig. S10a and b; Table S14), consistent with the characteristic aromatic profile of *Z. striolatum* rhizomes, in which monoterpenoids represent key volatile constituents [12, 13]. Notably, monoterpene biosynthetic genes in both subA and subB largely belonged to the TPS-b subfamily (Table S15), which is specialized for monoterpene synthase activity [39].

To further explore the evolutionary dynamics of the terpene synthase (TPS) gene family, we performed a comparative analysis across five Zingiberaceae species. *Z. striolatum* harbors 179 TPS genes, a count substantially exceeding those identified in *Z. officinale* (148), *R. debilis* (31), and *A. tsaoko* (37) (Fig. 3d). Phylogenetic analysis classified these TPS genes into seven subfamilies, with marked expansion of TPS-a and TPS-b in *Z. striolatum*, *Z. officinale*, and *C. longa* (Fig. 3d and e; Fig. S11a). This genomic expansion correlates with the substantial accumulation of monoterpenes and sesquiterpenes typically observed in their rhizomes. Notably, numerous TPS-a and TPS-b copies in *Z. striolatum* formed tandem clusters, suggesting that tandem duplication served as a primary driver of family expansion (Fig. S11b). Intriguingly, a Zingiberaceae-specific TPS lineage grouped within the TPS-d subfamily (marked in Fig. 3d and Fig. S11a), a group otherwise characteristic of gymnosperms and basal angiosperms and associated with the synthesis of monoterpenes, sesquiterpenes, and diterpenes [40]. This suggests that Zingiberaceae may have retained an ancient TPS-d-like lineage with functional properties distinct from conventional angiosperm TPS genes.

Gene Ontology (GO) enrichment indicated that expanded gene families were associated with oxidative stress response (GO:0006979), peroxidase activity (GO:0004601), and antioxidant activity (GO:0016209) (Fig. S12a and b; Table S16). These enrichments imply a reinforced reactive oxygen species (ROS) scavenging capacity that may facilitate resilience to UV exposure, low temperature, and humid environments [41]. In contrast, subB-specific contractions were enriched for Kyoto Encyclopedia of Genes and Genomes (KEGG) pathways associated with isoflavonoid (ko00943) and anthocyanin (ko00942) biosynthesis (Fig. S10c and d; Table S14). Anthocyanins support photoprotection against high light and UV-B [42–44], and contraction of their biosynthetic genes is consistent with the shaded, montane ecology of *Z. striolatum*. Isoflavonoids function as phytoalexins synthesized during pathogen challenge [45]; contraction of pathway genes therefore aligns with reduced pathogen pressure and limited symbiotic diversity at high elevations [46, 47]. Contracted gene families were also enriched for cell recognition (GO:0008037) (Fig. S12c and d; Table S16), with membrane receptors and glycosylation-based recognition [48, 49], further supporting adaptation to high-altitude habitats with reduced pathogen load.

### Identification of YRP components

*Z. striolatum* flower buds have gained attention due to their high natural pigment content [14]. To examine fertilizer effects on pigment composition and biosynthesis, the Guizhou cultivar ‘Ziyanghe’ (purplish-red buds, Pbu) was grown under three nutrient regimes: control (CK), high nitrogen–high potassium (HNK), and compound fertilizer (CF) (see Section ‘Methods’). The HNK treatment significantly enhanced productivity, yielding the highest bud number per hill, which represented 1.65- and 3.21-fold increases over the CF and CK, respectively (Fig. S13a). Specifically, fresh bud yields reached 33.0 t·hm^−2^ under HNK, surpassing 24.8 t·hm^−2^ in CF and 22.5 t·hm^−2^ in CK (Fig. S13b). To characterize pigment constituents and their molecular basis, comparative metabolomic and transcriptomic analyses were conducted on HNK-treated Pbu and white buds (Wbu) from the nonpigmented cultivar ‘Baiyanghe’.

A total of 29 216 metabolites were detected across Pbu and Wbu, of which 4516 showed differential abundance. Among these, 2776 were significantly upregulated and 1740 downregulated (Table S17). Flavonoids comprised 5.41% of differential metabolites (DMs) (Fig. 4a), and KEGG enrichment indicated involvement in flavone and flavonol biosynthesis (Fig. S14). Variable importance in projection (VIP > 1) identified 10 major DM contributors, including procyanidin A1, procyanidin A2, and quercetin as the most strongly elevated metabolites in Pbu (Fig. 4b; Table S17). Because anthocyanins represent the primary water-soluble pigments driving red-to-purple coloration in plant tissues [50], downstream analysis focused on anthocyanidin profiles.

**Figure 4:**
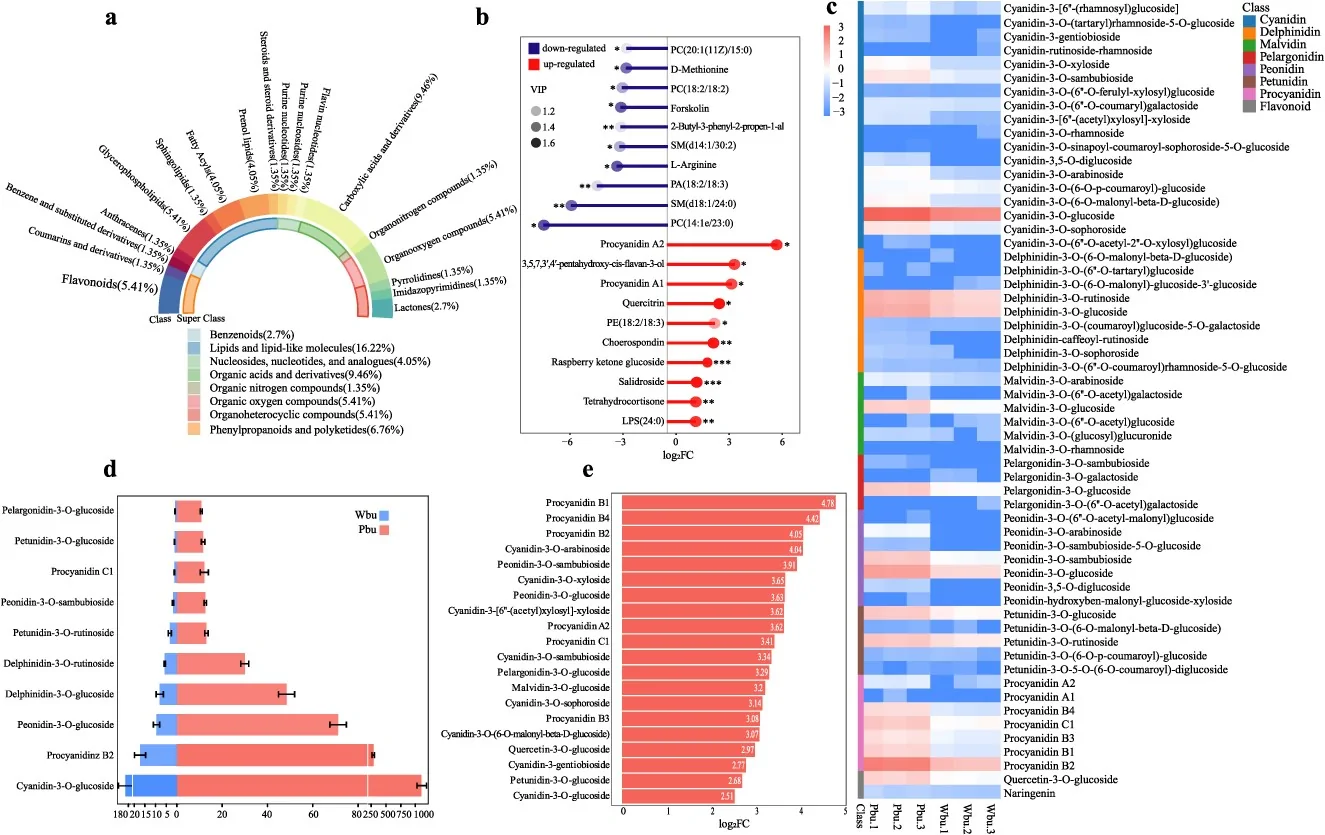
Metabolic profile of YRP. (a) Chemical classification of the metabolites identified in the flower buds of Pbu and Wbu. (b) Major contributors among DMs. (c) Heatmap of anthocyanin monomer expression in Pbu and Wbu. (d) Quantification of the primary anthocyanin pigment monomers identified in Pbu and Wbu. (e) Major contributors among ADMs.

LC–MS/MS profiling identified 49 anthocyanins and 9 non-anthocyanin flavonoids (Fig. 4c; Table S18). These included 18 cyanidins, 9 delphinidins, 7 peonidins, 6 malvidins, 5 petunidins, and 4 pelargonidins. The nine non-anthocyanin flavonoids comprised seven procyanidins, quercetin-3-O-glucoside, and naringenin. Most monomers accumulated at significantly higher levels in Pbu than in Wbu (Fig. 4d; Fig. S15). Cyanidin-3-O-glucoside was most abundant in both bud types, reaching 1091.5 μg/g in Pbu vs 191.05 μg/g in Wbu. Additional major compounds were procyanidin B2 (Pbu: 285.25 μg/g; Wbu: 17.16 μg/g), peonidin-3-O-glucoside (Pbu: 71.19 μg/g; Wbu: 5.74 μg/g), delphinidin-3-O-glucoside (Pbu: 48.44 μg/g; Wbu: 9.57 μg/g), and delphinidin-3-O-rutinoside (Pbu: 30.00 μg/g; Wbu: 8.04 μg/g).

To refine pigment composition, anthocyanin differential metabolites (ADMs) were screened using |Log2(FC)| ≥ 1. A total of 32 ADMs were identified, of which 29 accumulated more strongly in Pbu (Fig. 4e; Table S19). Thirteen monomers exceeded |Log2(FC)| ≥ 2.5, comprising eight cyanidins (cyanidin-3-O-arabinoside, cyanidin-3-O-xyloside, cyanidin-3-[6″-(acetyl)xylosyl]-xyloside, cyanidin-3-O-sambubioside, cyanidin-3-O-sophoroside, cyanidin-3-O-(6-O-malonyl-β-D-glucoside), cyanidin-3-gentiobioside, cyanidin-3-O-glucoside), two peonidins (peonidin-3-O-sambubioside, peonidin-3-O-glucoside), malvidin-3-O-glucoside, petunidin-3-O-glucoside, and pelargonidin-3-O-glucoside. Although delphinidin-3-O-glucoside and delphinidin-3-O-rutinoside showed |Log2(FC)| < 2.5, their absolute concentrations remained high. Quercetin-3-O-glucoside, while low in abundance (8.14 μg/g), showed a |Log2(FC)| of 2.97. As a flavonol co-pigment, quercetin-3-O-glucoside enhances anthocyanin color expression through hyperchromic and bathochromic effects, shifting pigments toward violet and improving color stability [51, 52]. In summary, cyanidin-3-O-glucoside, peonidin-3-O-glucoside, delphinidin-3-O-glucoside, and delphinidin-3-O-rutinoside represent major color-imparting pigments in *Z. striolatum* buds, while quercetin-3-O-glucoside functions as an enhancing co-pigment.

### Molecular insights into YRP biosynthesis

As illustrated in Fig. 5a, the anthocyanin pigments and the co-pigment quercetin that underlie Pbu coloration are synthesized in the cytoplasm via the phenylpropanoid, flavonoid, and anthocyanin pathways [20, 53]. Genome-wide identification of anthocyanin biosynthetic structural genes across five Zingiberaceae species and *Arabidopsis* yielded 167 candidate structural genes encoding 14 enzymes in *Z. striolatum* (Table S20). These genes were evenly distributed across subA, subB1, and subB2 (54, 60, and 54 genes), respectively (Fig. 5b). Compared with *Arabidopsis* and other ginger relatives (*R. debilis*, *A. tsaoko*, and *W. villosa*), *Z. striolatum* displayed gene family expansion, particularly within *phenylalanine ammonia-lyase* (*PAL*, 21 members), c*innamate 4-hydroxylase* (*C4H*, 16), *4-coumarate CoA ligase* (*4CL*, 26), *chalcone synthase* (*CHS*, 29), c*halcone isomerase* (*CHI*, 10), and f*lavonoid 3′,5′-hydroxylase* (*F3′5′H*, 30) (Fig. 5a). While most expanded copies were uniformly distributed across the three subgenomes, tandem duplication was specifically confined to *F3′5′H* and *PAL* (Fig. S16). Notably, 9, 14, and 7 *F3′5′H* copies were localized to subA, subB1, and subB2, respectively, forming dense tandem clusters on chromosomes 7, 8, and 9 (Fig. 5c).

**Figure 5:**
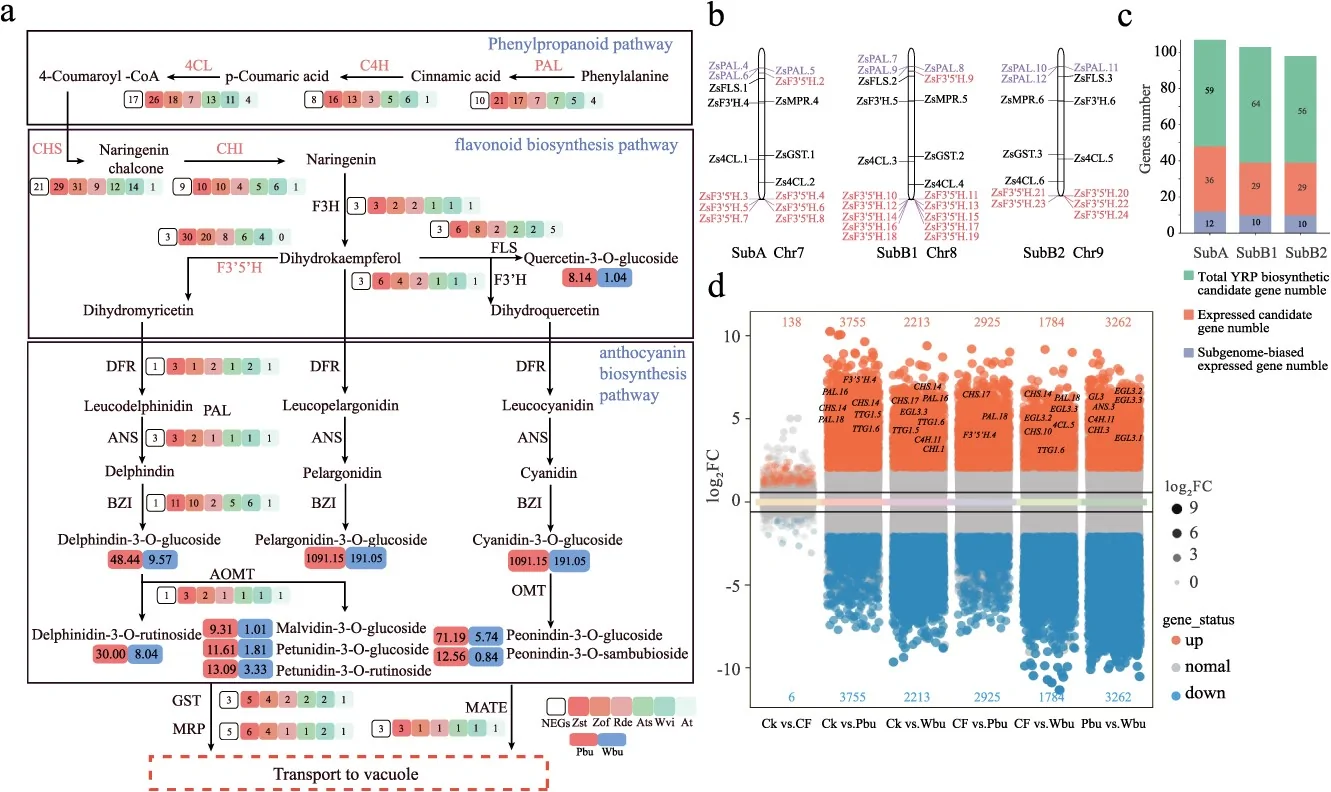
Molecular regulatory network of YRP biosynthesis. (a) Schematic representation of the metabolic and transcriptional regulation of the anthocyanin biosynthetic pathway. NEGs: number of expressed genes. Squares represent the number of genes in *Z. striolatum* (Zst), *Z. officinale* (Zof), *R. debilis* (Rde), *A. tsaoko* (Ats), *W. villosa* (Wvi), and *Arabidopsis* (At). Numbers within rectangles indicate anthocyanin monomer concentrations (μg/g) in Pbu and Wbu, respectively. (b) Chromosomal distribution of YRP biosynthesis candidate genes on chr07, 08, and 09. Notable tandemly duplicated clusters of *F3′5′H* genes are highlighted. (c) Distribution of YRP biosynthetic gene categories among the three subgenomes. (d) Statistical distribution of DEGs involved in YRP biosynthesis across fertilizer treatments (CK, HNK, and CF) and between the two bud phenotypes (Pbu vs Wbu).

Anthocyanins require sequestration in vacuoles following cytoplasmic synthesis, mediated by transporters including glutathione S-transferases (GSTs), multidrug and toxic compound extrusion (MATEs), and multidrug resistance-associated proteins (MRPs) [54, 55]. Accordingly, these gene families were characterized genome-wide. In total, 167 *ZstGSTs* clustered into 11 subfamilies, with notable expansion in tau and phi clades (Fig. S17), consistent with evolutionary patterns in other plants [56]. Five *ZstGSTs* were homologous to the *Arabidopsis* phi clade transporter *TRANSPARENT TESTA 19* (*TT19*), a well-known mediator of vacuolar anthocyanin transport [22]. Furthermore, we identified 113 MATE genes, among which three clade II members exhibited high homology to *TRANSPARENT TESTA 12* (*TT12*) (Fig. S18), a vacuolar transporter associated with proanthocyanidin and anthocyanin movement [57]. Similarly, the MRP family (also designated as the ABCC subfamily) was represented by 18 members (Fig. S19). Phylogenetic analysis revealed that six of these MRPs clustered closely with validated anthocyanin transporters: three grouped with maize *ZmMRP3* and grape *VvABCC1*, while three others exhibited high homology to *Arabidopsis AtABCC2* [58, 59]. These 14 core candidate transporters (five *GSTs*, three *MATEs*, and six *MRPs*) were uniformly distributed across the three subgenomes (Table S20), suggesting conserved regulatory and transport dosage across the allotriploid genome.

Transcriptomic profiling showed that 94 of 181 anthocyanin-related genes (167 structural and 14 transporters) were expressed in at least one treatment (Fig. 5b; Table S21): 36 in subA, 29 in subB1, and 29 in subB2. Expression patterns differed among treatments, with 35 genes most highly expressed under HNK (Pbu), 27 under CF, and 7 under Wbu, consistent with observed bud yield differences. The highly expressed genes under HNK and CF treatments encompassed all key enzymatic steps of the YRP biosynthesis pathway. Pearson correlation analysis identified 65 genes positively correlated with all measured metabolites (*P* < 0.05), with *Zst4CL.3* and *ZstCHI.2* showing the strongest statistical significance (*P* < 0.001). Key candidates, including *ZstF3′5′H.30*, *ZstF3′5′H.18*, *ZstFLS.2*, *ZstDFR.2*, *ZstANS.3*, *ZstAOMT.2*, *ZstAOMT.3*, *ZstMRP.4*, and *ZstGST.3*, displayed significant positive correlations with metabolite accumulation (*P* < 0.01) (Fig. S20).

To explore transcriptional regulation of YRP synthesis, 8450 transcription factors (TFs) from 64 families were identified (Table S22). Differential expression analysis across fertilizer treatments and between Pbu and Wbu revealed marked changes in MYB and bHLH families (Fig. S21). Among the upregulated TFs, we detected a MYB candidate (*ZstMYB123.2*) homologous to *Arabidopsis TRANSPARENT TESTA 2* (*TT2*), alongside four bHLH genes, including three *EGL* homologs (*ZstEGL3.1*, *ZstEGL3.2*, and *ZstEGL3.3*) and one *GL3* homolog (*ZstGL3*) (Fig. 5d; Table S23). Furthermore, three WD40 genes (*ZstTTG1.4*, *ZstTTG1.5*, and *ZstTTG1.6*) and core biosynthetic genes (*CHS*, *CHI*, *F3′5′H*, *ANS*, *AAT*, *GST*) were also detected (Table S23). Expression trends indicated strong upregulation of *ZstEGL* and *ZstGL3* in Pbu, whereas *ZstMYB123.2* showed higher expression in Wbu (Table S21). Further correlation analysis identified four candidate positive regulators (*ZstEGL3.1*, *ZstEGL3.2*, *ZstEGL3.3*, and *ZstTTG1.6*) and one negative regulator (*ZstMYB123.2*), significantly associated with metabolite accumulation (*P* < 0.05; Fig. S20). These findings suggest that while the bHLH and WD40 components likely function as positive regulators, *ZstMYB123.2* may play a distinct, potentially repressive role within the MBW-mediated regulatory landscape of *Z. striolatum*. Nevertheless, its precise molecular mechanism and functional involvement require further experimental validation.

### Subgenome-specific expression patterns and divergence of YRP biosynthetic genes

We characterized the homolog-specific expression patterns of genes involved in YRP biosynthesis to evaluate the transcriptional contributions of the three subgenomes. Overall, the global transcriptional output remained relatively balanced across the triploid genome, with subA, subB1, and subB2 contributing 36.20%, 33.35%, and 30.45%, respectively (Fig. 6a). This indicates a lack of strong subgenome dominance at the global level. To explore regulatory divergence after polyploidization, we identified 91 homologous gene pairs across 14 gene families (Table S24). Expression correlation analysis revealed that 73.6% (67 gene sets) maintained highly coordinated expression (*r* > 0.791), reflecting conserved transcriptional regulation across the pathway. Conversely, 15.4% (14 gene sets) exhibited significant expression divergence (Fig. 6b).

**Figure 6:**
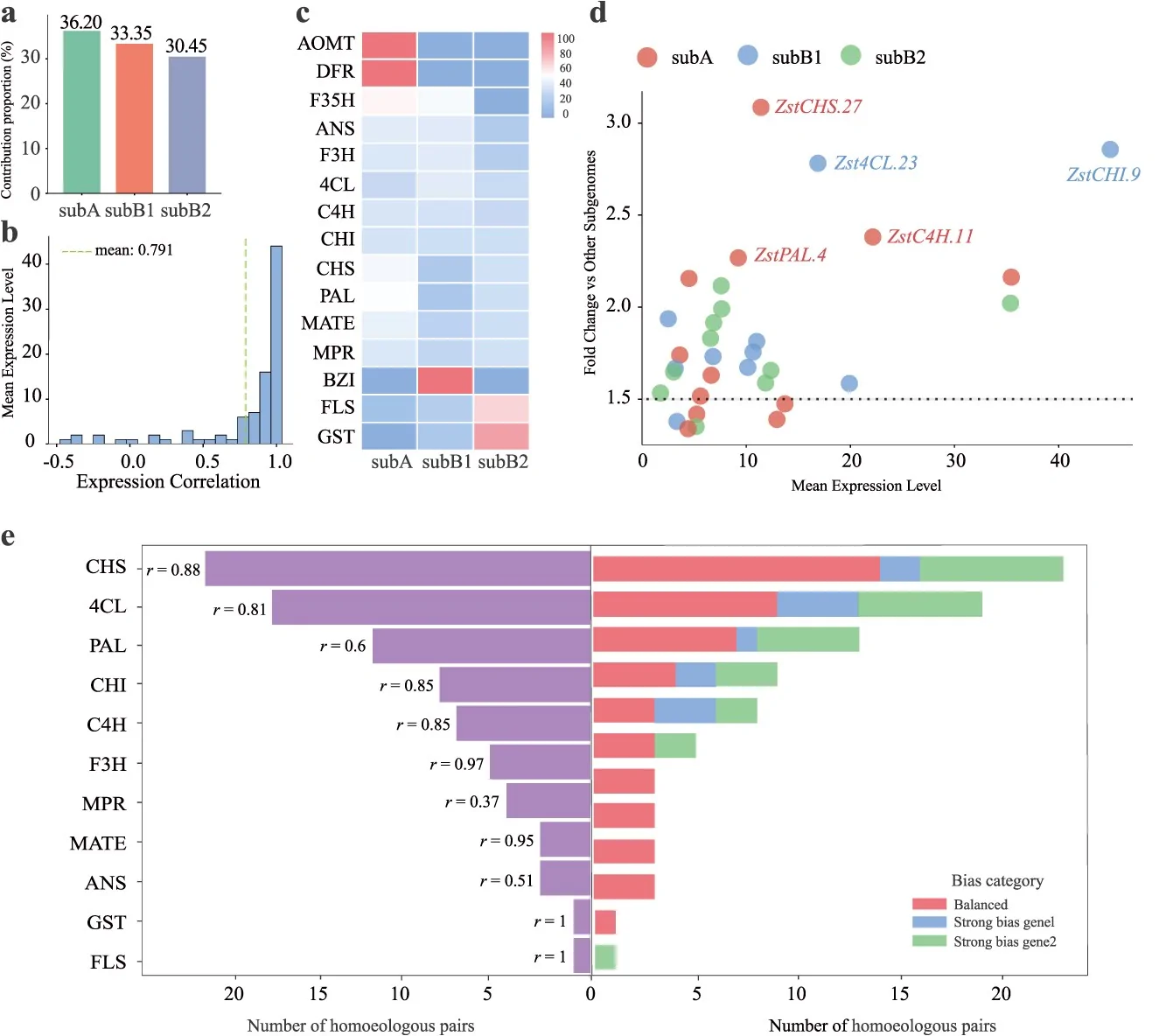
Subgenome expression bias analysis of YRP biosynthetic genes. (a) Proportional contribution of each subgenome to total transcript levels. (b) Distribution of expression correlation between homologous gene sets. (c) Heatmap showing subgenome expression bias categorized by gene family. (d) Subgenome-specific gene expression profiles. (e) Expression correlation (left) and subgenome expression bias (right) across different gene families.

Despite this global stability, specific key gene families displayed pronounced homolog expression bias (HEB). Notably, *AOMT* and *DFR* were exclusively expressed from subA, while *BZI* was contributed entirely by subB1 (Fig. 6c). Furthermore, genes involved in the upstream biosynthetic pathway, *PAL*, *4CL*, *CHS*, *CHI*, and *C4H*, displayed higher degrees of regulatory divergence and expression bias (Fig. 6d). For instance, *ZstPAL.4*, *ZstCHS.27*, and *ZstC4H.11* in subA; *Zst4CL.23* and *ZstCHI.9* in subB1; and *ZstCHI.10* in subB2 exhibited expression levels more than 2-fold higher than their respective homologs (Fig. 6e; Table S25). In contrast, core anthocyanin-specific genes (*F3H*, *F3′H*, *MATE*, *GST*) maintained high co-expression, likely ensuring the metabolic robustness of pigment production in the Pbu.

## Discussion

### High-fidelity assembly of the complex triploid *Z. striolatum* genome

The assembly of complex polyploid genomes with high heterozygosity and repetitive sequences is a major challenge in plant genomics. By integrating PacBio HiFi long-read sequencing with Hi-C scaffolding, this study produced the first chromosome-level, haplotype-resolved reference genome for the allotriploid *Z. striolatum* (7.97 Gb). The exceptional contig N50 (35.21 Mb) and LAI values (> 24) underscore the high structural continuity and assembly quality achieved. However, extreme sequence homology poses inherent technical challenges for Hi-C-based phasing, as ambiguous interaction signals can increase the risk of switch errors or chimeric scaffolds [60]. Although the HiFi of the *Z. striolatum* genome is supported by a minimal residual SNP rate (0.0053%) and uniform HiFi read coverage, minor phasing uncertainties may remain in repetitive or centromeric regions [61]. Future validation strategies, such as ultra-long nanopore sequencing or trio binning, may be necessary to further delineate the precise boundaries of these nearly identical haplotypes and advance toward an entirely error-free, telomere-to-telomere assembly [62].

### Evolutionary drivers of genome expansion and subgenomic asymmetry

The *Z. striolatum* genome possesses the most extensive repetitive content reported to date in Zingiberaceae, primarily driven by the rapid expansion of *Copia* and *Gypsy* LTR-RTs. The observed bursts of LTR activity at ~0.15 and 0.66 Mya align with the intense climatic oscillations of the late Pleistocene, suggesting that these TE eruptions may have represented a genomic response to environmental stressors [63]. Beyond driving physical genome size expansion, this LTR-mediated restructuring likely promoted functional diversification in key gene families by altering genomic landscapes and generating new *cis*-acting regulatory elements [63, 64]. These LTR bursts may have provided adaptive momentum for the ecological radiation of *Z. striolatum* across fluctuating environments. This process was likely built upon an ancestral Zingiberaceae-specific WGD event (~52.5 Mya), which provided the foundational genetic substrate for subsequent adaptive evolution [65].

Polyploidy serves as an evolutionary accelerator, with its establishment frequently coinciding with chromosomal restructuring [37, 48, 66–69]. *Z. striolatum* is an AAB-type triploid formed through allopolyploidy, with subgenomes A and B merging 1.2 Mya. Karyotype reconstruction reveals that the putative ancestral Zingiberaceae genome contained 12 chromosomes, and fusion of chr12 reduced the base number to 11 in the *Zingiber* lineage. In *Z. striolatum*, subB largely retains the ancestral configuration, punctuated by a single inversion, whereas subA has undergone more extensive remodeling characterized by three large-scale inversions. This asymmetry in chromosomal restructuring, coupled with the parallel subgenomic evolution of gene families associated with shade adaptation and volatile biosynthesis, underscores the complex interplay between polyploidy and niche specialization [70].

### Molecular coordination and subgenome-exclusive regulation of YRP biosynthesis

The distinctive purplish-red coloration of *Z. striolatum* flower buds is driven by the efficient accumulation of YRP [71]. Metabolomic profiling identified cyanidin-3-O-glucoside, peonidin-3-O-glucoside, delphinidin-3-O-glucoside, and delphinidin-3-O-rutinoside as the principal pigments, while quercetin-3-O-glucoside likely stabilizes these molecules through intermolecular co-pigmentation [72]. A total of 167 structural genes and 14 transporters associated with YRP biosynthesis were identified, most of which belonged to expanded gene families, providing a robust molecular framework for bud pigmentation. Among these, 94 genes were actively expressed. Although global transcriptional output appears balanced across the subgenomes (subA: 36.20%, subB1: 33.35%, and subB2: 30.45%), critical biosynthetic nodes exhibit pronounced regulatory divergence. Specifically, *AOMT* and *DFR* were expressed solely from subA. This subgenome-exclusive expression represents an extreme manifestation of HEB, potentially attributable to the pseudogenization or epigenetic silencing of corresponding homologs in the alternative subgenomes [73, 74].

Vacuolar accumulation, mediated by *GSTs*, *MRPs*, and *MATE* transporters, is essential for final color expression [75]. All *ANS* and *MATE* copies were highly expressed in Pbu, and the expression levels of *ZstDFR.2*, *ZsANS.3*, *ZstAOMT.2*, *ZstAOMT.3*, *ZstMRP.4*, and *ZstGST.3* displayed significant positive correlations with pigment abundance, suggesting that these genes likely serve as critical rate-limiting nodes in the anthocyanin biosynthetic pathway. Furthermore, the maintained high co-expression of *MATE* and *GST* across the three subgenomes likely ensures the metabolic robustness and stability of pigment production in the Pbu. The MBW transcriptional complex orchestrates anthocyanin biosynthesis by coordinating the expression of late biosynthetic genes, such as *DFR* and *ANS* [76, 77]. The high expression of *EGL3*, *GL3*, and *TTG1* homologs in Pbu, coupled with their strong correlation with metabolite levels, indicates that they are putative central regulatory hubs driving anthocyanin accumulation in *Z. striolatum*. Collectively, this study identifies key candidate genes for YRP biosynthesis through integrated multi-omics analysis and elucidates their regulatory roles. Future functional validation experiments will be needed to decipher the precise molecular mechanisms underpinning the coloration process.

## Materials and methods

### Plant materials and genome sequencing

The triploid *Z. striolatum* cultivar ‘Ziyanghe’, widely cultivated in Guizhou Province, China, was selected for whole-genome sequencing. Plant material was sourced from the germplasm nursery at Zunyi Normal University, Guizhou (106.92° E, 27.71° N). High-quality genomic DNA was extracted from young leaf tissue using a modified cetyltrimethylammonium bromide (CTAB) protocol. DNA concentration and purity were quantified using a Qubit fluorometer (Thermo Fisher Scientific, Waltham, MA, USA), and integrity was verified via agarose gel electrophoresis. High-quality DNA was then used to construct Illumina libraries with an insert size of 350 bp, generating 178.35 Gb of paired-end reads on the Illumina NovaSeq platform (Table S1).

To resolve the complexities of this large, highly heterozygous, and repeat-rich genome, long-read sequencing was performed. A standard SMRTbell library was constructed using the SMRTbell Express Template Prep Kit 2.0 (Pacific Biosciences, Menlo Park, CA, USA) according to the manufacturer’s instructions. DNA integrity was verified using an Agilent 2100 Bioanalyzer (RIN > 7.0). The library was sequenced on the PacBio Sequel II platform, yielding 78.45 Gb of long-read sequences with a read N50 of 17.45 kb (Table S1). For chromosome-level scaffolding, fresh young leaves were collected from clonally propagated seedlings of the same genotype and fixed with formaldehyde. Chromatin was digested with *DpnII* restriction enzyme (New England Biolabs, Ipswich, MA, USA), and Hi-C libraries were prepared following established protocols. Libraries meeting quality control standards were sequenced on the Illumina NovaSeq platform, yielding 326.92 Gb of raw sequence data (Table S1).

### Genome size estimation and haplotype-resolved reference genome assembly

Before assembly, the chromosome number of *Z. striolatum* was determined using the root-tip squash technique, while genomic features were estimated via flow cytometry and K-mer analysis. For cytogenetic characterization, healthy root tips were pretreated with 0.002 M 8-hydroxyquinoline at 25°C for 3 h, fixed in Carnoy’s solution (absolute ethanol:glacial acetic acid, 3:1, v/v) at 4°C for 2 h, and hydrolyzed in a 1:1 (v/v) mixture of 45% acetic acid and 1 M HCl at 60°C for 5 min. Following a 2-h hypotonic treatment, meristematic tissues were stained with modified carbol fuchsin and visualized using a LEICA DM1000 light microscope, confirming a chromosomal configuration of 2*n* = 3 *×* = 33.

Concurrently, nuclear DNA content was assessed via flow cytometry (BD FACScalibur) with maize (*Zea mays*) ‘B73’ (reference genome size: 2.3 Gb) as an internal standard, yielding an estimated genome size of 7.96 Gb for *Z. striolatum* (Fig. S1). Analysis of *K*-mer frequency distribution using GenomeScope 2.0 [78] revealed characteristic features of a triploid genome, with an estimated heterozygosity rate of 3.68%.

HiFi reads were generated using the CCS software (https://github.com/PacificBiosciences/ccs), and raw Hi-C reads were filtered using fastp v0.23.2 [79]. Subsequently, a *de novo* assembly was performed using hifiasm v0.16.1 [80] by integrating HiFi and Hi-C data in Hi-C mode. This yielded an 8.15 Gb contig-level genome, consisting of two primary components: a primary haplotype assembly (subA) representing a single subgenome and a larger assembly (subB) comprising unphased contigs derived from two subgenomes. Hi-C reads were aligned to these contigs using BWA-MEM v0.7.172 [81], followed by two iterations of error correction via NextPolish v1.1.03 [82]. Chromosome-scale scaffolding was achieved by clustering, ordering, and orienting polished contigs with ALLHiC v0.9.134 [83]. This strategy successfully resolved three distinct subgenomes (subA, subB1, and subB2) at chromosome-scale resolution.

To validate the quality of the *Z. striolatum* haplotype-resolved assembly, PacBio HiFi reads were aligned back to the genome to check for assembly collapse. Theoretically, unresolved homologous regions (assembly collapse) would result in localized HiFi read coverage significantly exceeding the genome-wide average. Our analysis revealed a uniform depth distribution consistent with the expected sequencing coverage, with no extensive or continuous regions of aberrant depth (Fig. S4), confirming that the three subgenomes were successfully resolved with minimal assembly collapse. Phasing precision was further validated by mapping Illumina reads to the final assembly using BWA [84], followed by SNP calling with GATK [85]. A low percentage of identified SNPs was used as a proxy for minimal residual heterozygosity, thereby confirming the accuracy of the triploid haplotype phasing (Table S4). Assembly completeness was confirmed and evaluated via BUSCO v5 [86] using the embryophyta_odb10 database. LAI was calculated using LTR_retriever v2.9.0 [87] to assess the assembly quality and continuity of intergenic and repeat-rich regions. Transcriptome unigenes were used as query sequences and were compared to the assembled genome using BLAT [88] to check the integrity of the assembled genome.

### Gene structure and function annotation

The genome annotation includes annotation of TEs and protein-coding genes. TEs in the *Z. striolatum* genome were identified by combining homology-based and *de novo* approaches. RepeatModeler [89] and LTRharvest [90] were used for *de novo* predictions, while RepeatMasker/RepeatProteinMask [91] were applied against the RepBase database [92]. Tandem Repeats Finder v4.09 [93] was used to detect tandem repeats. All results were integrated into a non-redundant repeat annotation dataset to calculate genomic proportions of different repeat types.

Protein-coding genes were predicted by integrating homology-based, *ab initio*, and transcriptome-assisted approaches. For homology-based prediction, protein sequences from six closely related species were aligned to the *Z. striolatum* genome using GeMoMa [94] to infer gene models. *Ab initio* prediction was performed using Augustus [95] and SNAP v2006-07-28 [96], both of which were trained on the assembled genome to enhance prediction accuracy. For transcriptome-assisted prediction, RNA-seq reads were aligned to the genome with HISAT2 v2.1.0 [97] and assembled into transcripts using StringTie v2.1.4 [98]. Open reading frames (ORFs) were then identified via TransDecoder v5.5.0. Simultaneously, Iso-Seq long-read data were aligned using Minimap2 [99] and further processed with PASA v2.4.1 [100] to refine full-length gene structures. Finally, these evidence-based models were integrated into a comprehensive consensus gene set using EVM v1.1.1 [100]. The quality of the final gene set was validated by assessing the overlap of CDS regions’ individual predictions and further evaluated for completeness using BUSCO v5 [86]. Functional annotation of the final gene set was performed by searching predicted protein sequences against the NCBI non-redundant (NR), Swiss-Prot, TrEMBL, KEGG, KOG, and InterPro databases using DIAMOND [101] with an *E*-value cutoff of 1e^−5^. The TFs were identified and classified using iTAK v18.12 [102], which encompasses reference data from 197 plant species, and were subsequently assigned to their respective gene families according to conserved domains.

### Identification and insertion timing of intact LTR-RTs

We quantified TEs’ evolutionary divergence in both subA and subB subgenomes of *Z. striolatum*. TE divergence was assessed by calculating PercDivs (percentage of substitutions in the matching region compared with the consensus) using RepeatMasker [91]. To identify intact LTR-RTs, we employed the LTR_retriever v2.9.0 to filter candidates initially predicted by LTR_finder and LTRharvest. Structural integrity was defined based on the presence of flanking LTRs with conserved 5′-TG. . .CA-3′ motifs, 4–6 bp target site duplications (TSDs), and internal functional motifs. Only elements with an internal distance between 100 bp and 15 kb were retained for subsequent analysis.

For these intact LTR-RTs, the insertion time (*T*) was estimated based on the sequence divergence (*K*) between the 5′ and 3′ LTRs using the formula *T* = *K*/(2*r*). Following the parameters established by Ma and Bennetzen [103], a substitution rate (*r*) of 1.3e^−8^ substitutions per site per year was adopted as the molecular clock. This chronological analysis provides critical insights into the temporal dynamics of LTR bursts, their contribution to genomic architecture, and the subsequent subgenomic divergence and polyploid consolidation of the triploid accession.

### Genome evolution analysis

To reconstruct the evolutionary history of *Z. striolatum*, we analyzed the protein-coding gene sets of its subgenomes (A and B subgenomes) alongside 17 representative plant species. This comparative dataset included 2 ANA-grade angiosperms (*Amborella trichopoda*; *Nymphaea colorata*), 1 eudicot (*A. thaliana*), and 11 monocots. The monocot lineage included the early-diverging orchid *Apostasia shenzhenica*, as well as *Phoenix dactylifera*, *Ananas comosus*, *Z. mays*, *O. sativa, M. acuminata*, and six additional Zingiberaceae species. All protein sequences were filtered to retain the longest transcript for each gene. An all-vs-all comparison was performed using BLASTP with an *E*-value cutoff of 1e^−5^. Orthologous groups were subsequently identified using OrthoMCL v1.4 [104] with an inflation parameter of 1.5.

We identified 264 single-copy orthologous families for phylogenetic reconstruction. Multiple sequence alignments were conducted using MUSCLE v3.8.31 [105]. A maximum likelihood phylogenetic tree was inferred using RAxML v8.2.12 [106] under the GTRGAMMA substitution model with1000 bootstrap replicates. Divergence times were estimated using the MCMCTREE program within the PAML package v4.7 [107]. We employed the independent rates model (clock = 2) and the GTR substitution model. Four calibration points sourced from the TimeTree database were used as constraints (*A. comosus* vs *O. sativa*: 94.1–117.0 Mya; *A. trichopoda* vs *O. sativa*: 179.9–205.0 Mya; *A. thaliana* vs *O. sativa*: 142.1–163.5 Mya; *M. acuminata* vs *Z. officinale*: 50.6–91.1 Mya). The MCMC process was run for 2 000 000 generations after a 200 000-generation burn-in to ensure parameter convergence.

### Gene family expansion and contraction analysis

Orthologous gene families were constructed using OrthoFinder v2.5.4 with default parameters (*E*-value < 10^−5^, sequence alignment coverage ≥ 30%) [108]. To avoid bias in evolutionary rate estimation, gene families with extreme copy numbers (≤3 or ≥ 1000 members in any single species) were excluded from subsequent analyses. The expansion and contraction of these families were subsequently inferred using CAFÉ v4.2 [109] based on a stochastic birth–death model. Utilizing the time-calibrated phylogenetic tree as a framework, we estimated a single global birth–death rate (λ) for all lineages through maximum likelihood estimation. Gene families with a conditional *P*-value < 0.05 were defined as significantly expanded or contracted. KEGG functional enrichment analysis was performed on the significantly expanded and contracted gene families by using the clusterProfiler R package [110].

### Collinearity and WGD analysis

Intragenomic and interspecific syntenic blocks were identified using the Python version of MCscan v 0.8 [111]. For intragenomic analysis, collinear blocks were identified between the subgenomes of *Z. striolatum* to detect polyploidization events. For interspecific comparisons, BLASTP searches (*E* -value < 1e^−5^) were employed to identify reciprocal best-hit (RBH) gene pairs between *Z. striolatum* and its close relatives (*Z. officinale*, *R. debilis*, and *A. tsaoko*). The number of syntenic blocks and gene pairs was quantified to assess the conservation of gene order and functional genomic architecture.

To estimate the timing of divergence and WGD events, we calculated the *Ks* for collinear gene pairs. Protein sequences within syntenic blocks were first aligned using ClustalW, and the resulting alignments were converted into codon-based nucleotide alignments. *Ks* values were subsequently calculated using the *codeml* program of PAML [107] under the F3X4 model. The absolute evolutionary rate (*r*) was determined using the formula *r* = *Ks*/2 *T*, where *Ks* is the median value of orthologous pairs and *T* is the divergence time derived from our calibrated phylogenetic tree. Finally, the WGD timing was inferred by dividing the *Ks* peak value of paralogous blocks by 2*r*.

### Comparative subgenome structural analysis

To characterize the structural divergence among the three subgenomes of *Z. striolatum*, chromosome-scale assemblies for each of the homologous linkage groups (LGs) were subjected to pairwise alignment using Minimap2 v2.x with the -x asm20 parameter [99]. These subgenomic sequences were then aligned against the *Z. officinale* genome, which served as a diploid reference framework. The resulting primary alignments were filtered and processed via SyRI v1.6.3 [112] with -k -F S flags to identify: (i) macrosyntenic blocks, (ii) single-nucleotide variants (SNVs), and (iii) SVs, including inversions, translocations, and duplications.

### Karyotype evolution of *Zingiber*

Ancestral karyotype reconstruction and chromosomal rearrangements were analyzed using MCScan and WGDI v0.6.5 by identifying syntenic blocks and conserved gene order. The ancestral Zingiberaceae genome (*x* = 12) was reconstructed to infer evolutionary trajectories toward the proto-Zingiber (*x* = 11) and extant lineages. Rearrangements, including translocations, inversions, and the fusion of ancestral chromosome 12, were quantified by comparing the *Z. striolatum* subgenomes (subA and subB) against ancestral configurations to assess structural stability and identify lineage-specific events.

### Fertilization treatment experiment

The field experiment was conducted in Xionshi Village, Yanzikou Town, Bijie City, Guizhou Province, China (105.42°E, 27.59°N). The experimental site features sandy loam soil with an organic matter content of 4.2% and a pH of 6.8. The experiment employed a randomized plot design with three fertilization treatments, each established in 32.4 m^2^ plots. Three fertilization regimes were tested: (i) Control (CK) with no topdressing applied throughout the growth period; (ii) a staged HNK treatment consisting of an initial application of high-nitrogen (high-N) fertilizer (N:P_2_O_5_:K_2_O = 22:8:10), followed by a high-potassium (high-K) fertilizer (13:7:38); and (iii) a compound fertilizer (CF) treatment involving two sequential applications of a balanced compound fertilizer (15:15:15).

Disease-free tubers were planted in February 2024, with topdressings applied on April 25 and May 16, 2024 (1.4 kg/plot per application, equivalent to 432 kg·ha^−1^). At harvest (7 August 2024), three randomized 5-m^2^ quadrats per plot were sampled for flower-bud count per plant and yield assessment. The results showed that the yield and quantity of flower buds treated with HNK were significantly higher than those of the other two treatments.

### Determination of YRP components and composition using LC–MS/MS

To determine the components and composition of YRP, Pbu from the HNK (Pbu) treatment and the Wbu from the local ‘Baiyanghe’ variety (Wbu) were subjected to LC–MS/MS analysis. Freeze-dried samples were pulverized (30 Hz, 1.5 min) and extracted with methanol/water/HCl (500:500:1 v/v/v; 0.5 ml per 0.05 mg powder). Following vortexing (5 min), ultrasonication (5 min), and centrifugation (12 000 rpm, 4°C, 3 min; 5424R, Eppendorf), supernatants were filtered (0.22-μm nylon membrane) and analyzed using UPLC-ESI-MS/MS (ExionLC™ AD & Q-Trap 6500+, SCIEX). Separation occurred on a BEH C18 column (Waters; 1.7 μm, 2.1 × 100 mm, 40°C) with 0.1% formic acid in water (A) and methanol (B) at 0.35 ml/min: 95% A (0 min), linear gradient to 5% A (12 min), hold 2 min, and re-equilibration to 95% A (14–16 min). MS parameters: ESI+ mode (5500 V), 550°C source temperature, and 35 psi curtain gas. Metabolites were identified via scheduled MRM using standard-curated MWDB (Metware), with declustering potential (DP) and collision energy (CE) optimized per compound. Quantitation employed Multiquant 3.0.3 (SCIEX) using characteristic fragment ions and triple-filtered spectra to eliminate non-target interference.

### Identification of candidate genes for the YRP biosynthesis

Putative anthocyanin biosynthesis homologs in *Z. striolatum*, *Z. officinale*, *R. debilis*, *A. tsaoko*, *W. villosa*, and *C. longa* were identified by BLASTP searches using *Arabidopsis* reference genes (*E*-value ≤ 1e^−10^). Multiple sequence alignments were generated with MAFFT v7.4712 [113], followed by automated trimming with trimAl v1.13 [114] under default parameters. Maximum-likelihood phylogenies were reconstructed using FastTree v2.1.114 (http://www.microbesonline.org/fasttree/) with 1000 bootstrap replicates and visualized in iTOL (https://itol.embl.de/) [115].

To investigate subgenome expression bias among the identified YRP biosynthetic candidate genes, homologous gene sets (triplets) distributed across the subA, subB1, and subB2 subgenomes were selected for comparative analysis. Paired *t*-tests were performed to detect biased expression between homologs across these subgenomes. Significant expression bias was defined using a threshold of *P* < 0.01 and FDR < 0.05, requiring at least a 2-fold difference in average expression levels.

### Transcriptome data analysis

To characterize the expression profiles of YRP biosynthetic genes, RNA-seq was performed on flower buds from three fertilization treatments (CK, HNK, and CF) and the native white-bud variety (Wbu), with three independent biological replicates per group. Total RNA was extracted using the BioMarker Plant RNA Kit (Beijing, China), with RNA concentration and integrity verified by NanoDrop spectrophotometry (Thermo Fisher Scientific) and Agilent 2100 Bioanalyzer, respectively. Sequencing libraries were constructed using the TruSeq Stranded mRNA kit (Illumina, San Diego, CA, USA) and sequenced on an Illumina NovaSeq 6000 platform, generating 150-bp paired-end reads.

Raw sequencing reads were quality-filtered and adapter-trimmed using Cutadapt v1.18 [116]. The resulting clean reads were aligned to the *Z. striolatum* reference genome using HISAT2 v2.2.1 [97] with default parameters. Read counts were calculated with featureCounts v2.0.1 [117] using SAM results from HISAT2, and FPKM (fragments per kilobase of transcript per million mapped reads) values were then calculated for every gene in the samples. The differentially expressed genes (DEGs) were identified using the DESeq2 R package v1.30.0 [118] based on |log_2_ (fold-change)| ≥ 1 and adjusted *P* value (*P* ≤ 0.05).

## Supplementary Material

Web_Material_uhag214

## Data Availability

All genomic and transcriptomic datasets generated in this study have been deposited in the National Genomics Data Center (NGDC) [119]. Raw sequencing data are archived in the Genome Sequence Archive (GSA, https://ngdc.cncb.ac.cn/gsa) [120] under accession numbers CRA039666 and CRA039586. The genome assembly and annotation files are available at figshare (https://doi.org/10.6084/m9.figshare.31908493).
