# Supplementary material for: A haplotype-resolved triploid genome assembly of Zingiber striolatum sheds light on genome expansion and trait evolution in Zingiberaceae
Source: Hortic Res. 2026 Jun 3;13(11):uhag214. doi: 10.1093/hr/uhag214 (PMC13719175; doi:10.1093/hr/uhag214)
Supplement: Supplementary file 1 [file web_material_uhag214.zip › Supplement Figure.docx]

**Supplement Figure**

**A haplotype-resolved triploid genome assembly of Zingiber striolatum sheds light on genome expansion and trait evolution in Zingiberaceae**

**Supplementary Fig. S1 Determination of genome size and chromosomal configuration in *Z. striolatum*. a.** Estimation of genome size and ploidy level in *Z. striolatum* via flow cytometry. Maize (Zea mays ‘B73’) was utilized as the internal standard (P2) with a known 2C DNA content of 5.43 pg, while P3 represents the *Z. striolatum* sample. The mean fluorescence intensities of P2 and P3 were 2316 and 3474, respectively. The genome size of *Z. striolatum* was calculated as: (3474 / 2316) × 5.43 pg × 978 Mb/pg ≈ 7965.81 Mb.**b**. Representative micrograph of *Z. striolatum* chromosome spreads obtained via the root-tip squash technique.

**Supplementary Fig. S2** Hi-C contact matrix of the 33 chromosomes in the *Z. striolatum* genome. The vertical color bar on the right represents the log_2_-transformed interaction frequency between genomic loci, with increased intensity indicating higher contact density.

**Supplementary Fig. S3** Subgenome construction. The 33 pseudomolecules of *Z. striolatum* were divided into two subgenomes: subA comprises Chr01, 04, 07, 10, 13, 17, 21, 24, 25, 30, and 31; subB was further partitioned into two subgroups, subB1 (Chr02, Chr05, Chr08, Chr11, Chr14, Chr16, Chr19, Chr22, Chr26, Chr28, and Chr32) and subB2 (Chr03, Chr06, Chr09, Chr12, Chr15, Chr18, Chr20, Chr23, Chr27, Chr29, and Chr33). The color bar at the top indicates the *Z*-score-normalized relative abundance of *K*-mers, where higher *Z*-scores represent an increased relative frequency of specific *K*-mer signatures within each subgenome.

**Supplementary Fig. S4** Genomic distribution of PacBio HiFi read coverage across the 33 *Z. striolatum* pseudochromosomes.

**Supplementary Fig. S5** Statistics of prediction results of gene structure of *Z. striolatum* and other species. **a**. Gene length; **b**. Exon length; **c**. CDS length; **d**. Intron length; **e**. Exon number.

**Supplementary Fig. S6** Functional annotation of protein-coding genes in *Z. striolatum*. **a**. Venn diagram illustrating the distribution and overlap of functional annotations across five primary databases: NR, InterPro, KEGG, SwissProt and KOG; **b**. Functional classification of predicted genes based on the KOG; **c**. Pathway distribution and classification of annotated genes according to the KEGG database; **d**. GO classification of the annotated genes, categorized into Biological Process, Molecular Function, and Cellular Component.

**Supplementary Fig. S7** Synteny analysis among *Z. striolatum* subgenomes.

**Supplementary Fig. S8** Comparative synteny dot plots between the triploid *Z. striolatum* (Zst) and representative diploid Zingiberaceae species. Each dot represents a collinear gene pair identified between the genomes. The panels illustrate a clear 3:1 syntenic ration between the triploid *Z. striolatum* genome and its diploid relatives: *Z. officinale* (Zsof) (**a**), *R. debilis* (Rde)(**b**), *A. tsaoko* (Ast)(**c**), *W. villosa* (Wvi)(**d**)

**Supplementary Fig. S9** Comparative genomic analysis among 18 plant species. **a**. Comparison of the number of gene families between the genomes of 18 plant species. **b**.Venn diagram shows gene families of *Z. striolatum*, Z. officinale, *R. debilis*, and W. villosa.

**Supplementary Fig. S10** KEGG pathway enrichment analysis of significantly expanded and contracted gene families in *Z. striolatum*. **a** and **b** Enrichment of significantly expanded gene families in subA and subB, respectively; **c** and **d** Enrichment of significantly contracted gene families in subA and subB, respectively.

**Supplementary Fig. S11** TPS genes of *Z. striolatum*. **a**. Phylogenetic analysis of *Z. striolatum* and seven representative plant TPS gene families. **b**. Chromosome distributions of TPS genes.

**Supplementary Fig. S12** GO pathway enrichment analysis of significantly expanded and contracted gene families in *Z. striolatum*. **a** and **b** Enrichment of significantly expanded gene families in subA and subB, respectively; **c** and **d** Enrichment of significantly contracted gene families in subA and subB, respectively.

**Supplementary Fig. S13** Quantification of flower bud yield in purplish-red bud *Z. striolatum* under three fertilization treatments. **a**. Average number of flower buds per hill. **b**. Average yield per plot.

**Supplementary Fig. S14** KEGG pathway enrichment analysis of differential metabolites in Pbu (purplish buds) and Wbu (white buds).

**Supplementary Fig. S15** Quantification of individual anthocyanin components in Pbu and Wbu.

**Supplementary Fig. S16** Chromosomal distribution of YRP biosynthesis-related candidate genes in triploid *Z. striolatum* genome.

**Supplementary Fig. S17** Phylogenetic characterization of GST genes across eight plants. A total of 167 *Z. striolatum* GST genes (ZstGSTs) were identified and categorized into 11 subfamilies. Significant gene family expansion was observed within the *tau* and *phi* subfamilies. Red stars denote the *Arabidopsis* reference gene *AtGSTF12* (*TT19*). Notably, *Z. striolatum* possesses five TT19 homologs.

**Supplementary Fig. S18** Phylogenetic characterization of the MATE transporter gene family across seven plant species. A total of 13 MATE genes were identified in *Z. striolatum* (ZstMATEs) and categorized into five distinct subfamilies. The *Arabidopsis* reference gene *TT12*, a well-characterized vacuolar transporter for proanthocyanidins and anthocyanins, clustered within Clade II. This clade contains three *ZstMATE* orthologs of *TT12*.

**Supplementary Fig. S19** Phylogenetic characterization of the MRP (ABCC) transporter gene family. Reference proteins known to mediate anthocyanin vacuolar transport, including *AtABCC2* from *Arabidopsis*, *ZmMRP3* from *Z. mays*, and *VvABCC1* from *Vitis vinifera*, are highlighted to provide functional context. A total of 18 MRP genes were identified in *Z. striolatum* (ZstMRPs). Among these, three *ZstMRP* orthologs clustered with the *ZmMRP3* and *VvABCC1* clade, while another three were identified as putative orthologs of *AtABCC2*, marking them as key candidates for anthocyanin sequestration in *Z. striolatum*.

**Supplementary Fig. S20** Correlation analysis between anthocyanin biosynthetic gene expression and anthocyanin accumulation. The heat map illustrates the Pearson correlation between the expression profiles of candidate anthocyanin biosynthetic genes and the relative anthocyanin content in Pbu and Wbu. * indicates *p*_value < 0.05; ** indicates *p*_value < 0.01; *** indicates *p*_value < 0.001.

**Supplementary Fig. S21** Number of differentially expressed transcription factors across three fertilization treatments and between Pbu and Wbu .
